# IntOMICS: A Bayesian Framework for Reconstructing Regulatory Networks Using Multi-Omics Data

**DOI:** 10.1089/cmb.2022.0149

**Published:** 2023-05-10

**Authors:** Anna Pačínková, Vlad Popovici

**Affiliations:** ^1^ RECETOX, Faculty of Science, Masaryk University, Brno, Czech Republic.; ^2^ Faculty of Informatics, Masaryk University, Brno, Czech Republic.

**Keywords:** Bayesian networks, integrative analysis, multi-omics, regulatory network

## Abstract

Integration of multi-*omics* data can provide a more complex view of the biological system consisting of different interconnected molecular components. We present a new comprehensive R/Bioconductor-package, IntOMICS, which implements a Bayesian framework for multi-*omics* data integration. IntOMICS adopts a Markov Chain Monte Carlo sampling scheme to systematically analyze gene expression, copy number variation, DNA methylation, and biological prior knowledge to infer regulatory networks. The unique feature of IntOMICS is an *empirical* biological knowledge estimation from the available experimental data, which complements the missing biological prior knowledge. IntOMICS has the potential to be a powerful resource for exploratory systems biology.

## 1. INTRODUCTION

Multi-*omics* data collect multiple modalities from the same set of samples and describe different aspects of cellular functioning. Integrative analysis combining multi-*omics* data can enhance our understanding of biological systems consisting of interconnected molecular components, which is crucial for developing novel personalized therapeutic strategies for complex diseases. Therefore, developing a freely available and user-friendly computational framework to infer regulatory relationships by integrating multiple *omics* data is one of the most relevant problems in systems biology (Hasin et al., 2017; Subramanian et al., 2020; Kang et al., 2022). Bayesian networks (BNs) are models used to represent probabilistic relationships between multiple interacting entities (Pearl, 1988; Cooper, 1989; Neapolitan, 1990). Over the past decades, BNs have become popular in computational biology (Lucas et al., 2004).

We present a new comprehensive R package, IntOMICS—a Bayesian framework based on Markov Chain Monte Carlo (MCMC) (Madigan et al., 1995) for multi-*omics* data integration, which combines prior knowledge with data-derived evidence for inferring regulatory networks. IntOMICS complements the missing prior knowledge using *empirical* biological knowledge estimated from the available experimental data. For further details about the IntOMICS algorithm, its performance and benchmark analysis, see Pačínková and Popovici (2022). IntOMICS implementation also includes functions to visualize *empirical* biological knowledge and generate diagnostic plots of an MCMC sampling scheme (Madigan et al., 1995).

## 2. DESIGN AND IMPLEMENTATION

**FIG. 1.**
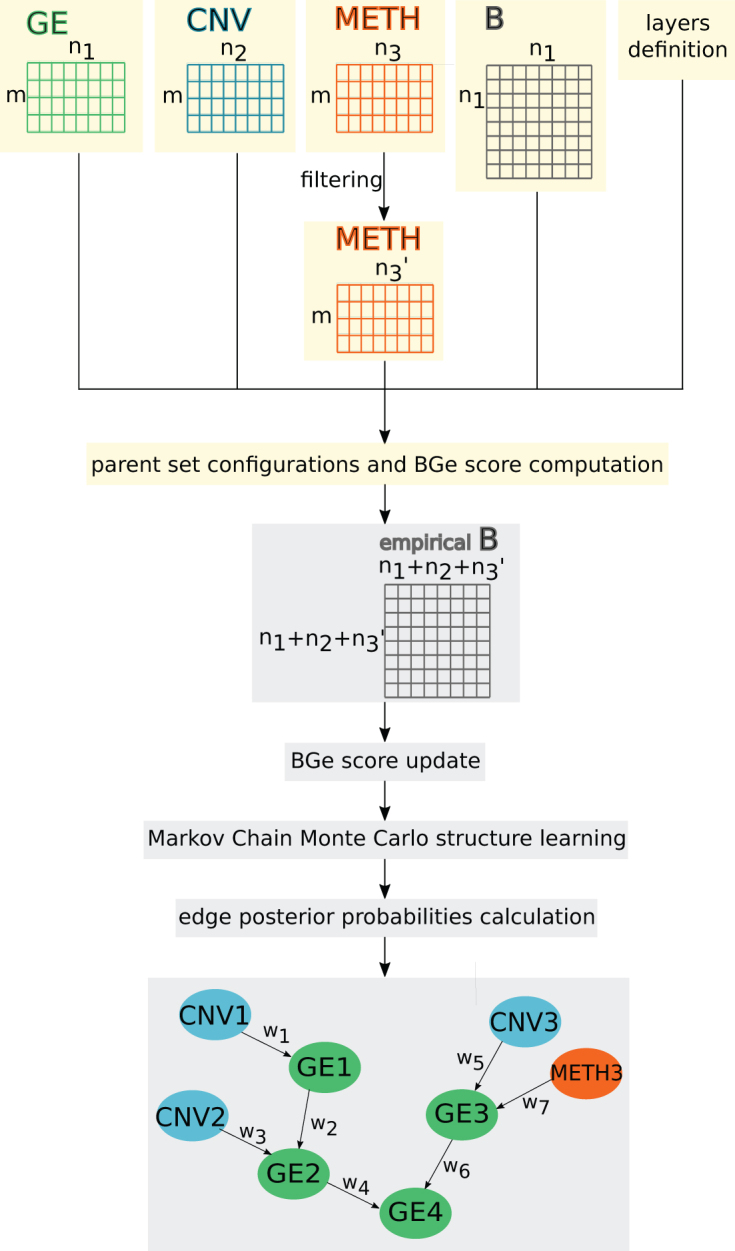
IntOMICS workflow. IntOMICS consists of two independent modules: yellow nodes are part of the *OMICS module* and gray nodes are part of the *BN module*. Edge weight *wi* represents the empirical frequency of given edge over samples of network structures. B, biological knowledge; BN, Bayesian network; CNV, copy number variation; GE, gene expression; METH, methylation.

IntOMICS implementation consists of two modules (Fig. 1). The *OMICS module* includes data preprocessing and computing some quantities needed to score a BN. IntOMICS apply the BGe score (Geiger and Heckerman, 1994) developed for continuous data. The *BN module* includes the MCMC sampling scheme for structure learning and sampling of BNs. In the last part of the *BN module*, IntOMICS infers the resulting network structure, including the edge weights representing the empirical frequency of given edges over the sample of network structures.

Input to IntOMICS are

- data matrices that represent collections of features for a set of samples (gene expression matrix [*GE*], copy number variation matrix [*CNV*], and DNA methylation matrix [*METH*]) and
- biological prior knowledge, which contains the information on known interactions among molecular features from public database(s).

IntOMICS is designed to infer regulatory networks, even if copy number variation or DNA methylation data (or both) are not available.

IntOMICS adapts MCMC scheme to multi-*omics* data—GE, CNV, and METH—by layers definition. Edges from the GE to the CNV/METH layers are excluded from the set of candidate edges. The resulting regulatory network structure consists of three types of nodes: GE nodes refer to gene expression levels, CNV nodes refer to copy number variations, and METH nodes refer to DNA methylation levels. Edge weight *w_i_* represents the empirical frequency of a given edge over samples of network structures.

Although the method is designed to work on any modalities defined in a continuous domain, the current implementation is tuned for gene expression, copy number variation, and DNA methylation. Adding a new modality requires the implementation of a new interface for the *OMICS module*, whereas the computational engine in the *BN module* remains the same. In that case, the *OMICS module* interface needs to be modified to capture all possible regulators of nodes from the given layer and accordingly define all possible parent set configurations.

### 2.1. Usage example

We use IntOMICS to investigate Wnt signaling and the role of the *FOXM1* gene in epithelial ovarian cancer (EOC) using 17 samples from the GSE146556 data set (Zhang et al., 2020) consisting of GE, CNV, and METH data. EOC is characterized by TP53 mutations, DNA copy number aberrations, numerous promoter methylation events, and *NOTCH* and *FOXM1* signaling activation (The Cancer Genome Atlas Research Network, 2011). *FOXM1*, one of the crucial oncogene drivers of EOC proliferation, is upregulated in EOC (The Cancer Genome Atlas Research Network, 2011; Zhang et al., 2020).

Chen et al. (2016) identified *FOXM1* as a novel target of the *Wnt* signaling essential for β-*catenin* activation. *FOXM1* accumulation in the nucleus promotes activation of *Wnt* signaling pathway by protecting the β-*catenin/TCF4* complex from inhibition by *CTNNBIP1*. *USP5–FOXM1* association abolishes the *CTNNBIP1* inhibition of the β-*catenin*/*TCF4* complex. *GSK3* activity enhances *FBXW7*-mediated *FOXM1* ubiquitination resulting in protein degradation. We select 14 genes from the Kyoto Encyclopedia of Genes and Genomes (Ogata et al., 1999) *Wnt* signaling pathway together with *FOXM1*, *USP5*, and *FBXW7* genes to infer the regulatory network using IntOMICS.

The first step is to perform data preprocessing and compute quantities needed to score a BN using omics_module() function:

> OMICS_mod_res <- omics_module(omics = omics, PK = PK, layers_def = layers_def, TFtargs = TFtarg_mat, annot = annot, gene_annot = gene_annot, lm_METH = TRUE, r_squared_thres = 0.5)

It is possible to use linear regression to filter irrelevant DNA methylation probes through lm_METH = TRUE. Arguments such as r_squared_thres or p_val_thres can be used to define the minimal *R*^2^ or the *p*-value threshold to determine a significant result.

The next step is to estimate model parameters and generate a sample of BNs from posterior distribution:

> BN_mod_res <- bn_module(burn_in = 100000, thin = 500, OMICS_mod_res = OMICS_mod_res, minseglen = 50000)

Now we can generate the diagnostic plots of the MCMC simulation and filter the most reliable edges in the resulting network structure (in this example, we use 0.75 quantile of all edge weights as the edge weight threshold):

> trace_plots(mcmc_res = BN_mod_res, burn_in = 10000, thin = 500, edge_freq_thres = 0.75)

> res_weighted <- edge_weights(mcmc_res = BN_mod_res, burn_in = 10000, thin = 500, edge_freq_thres = 0.5)

> weighted_net_res <- weighted_net(cpdag_weights = res_weighted, gene_annot = gene_annot, PK = PK, OMICS_mod_res = OMICS_mod_res, gene_ID = “gene_symbol,” TFtargs = TFtarg_mat, B_prior_mat_weighted = B_prior_mat_weighted(BN_mod_res))

ggraph_weighted_net() function is used to visualize the resulting network structure with the color scale for all modalities used in the network structure inference:

> ggraph_weighted_net(net = weighted_net_res)

**FIG. 2.**
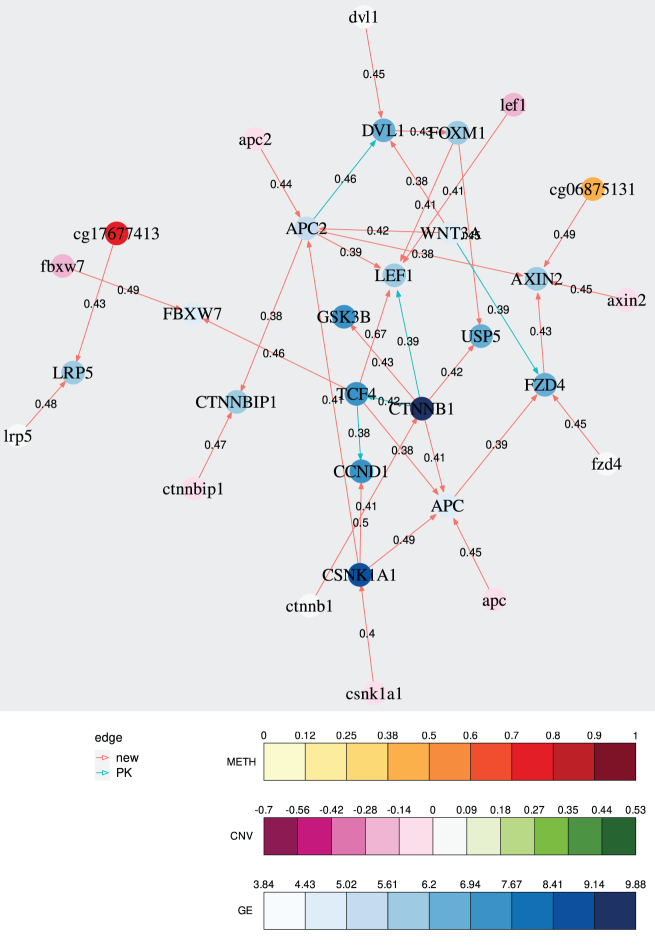
Example of the IntOMICS output. GE features are denoted by upper case, CNV features are denoted by lower case, and DNA METH features are denoted by methylation probe names (cgxxxx). PK, prior knowledge.

The resulting regulatory network can be seen in Figure 2. We can see several interactions known from the biological prior knowledge, including interactions from *CTNNB1* (β-*catenin*) to *TCF4* and from *TCF4* to *CCND1*. IntOMICS also identified the interaction between *USP5* and *FOXM1*. On the contrary, the interaction from *CTNNBIP1* to *CTNNB1* is not identified. CNV associated with GE is identified in several genes, including tumor suppressor *FBXW7*. Some of them were identified as significantly deleted in ovarian cancer, such as *LEF1* or *CTNNBIP1* (The Cancer Genome Atlas Research Network, 2011). METH-GE interactions were identified in *AXIN2* and *LRP5* (previously reported as hypermethylated in EOC; Dai et al., 2011). These results suggest IntOMICS identified interactions expected to be observed in EOC samples with *FOXM1* overexpression.

## 3. CONCLUSION

We present IntOMICS as a comprehensive and powerful tool for regulatory network inference using multi-*omics* data. IntOMICS combines prior knowledge with data-derived evidence to advance regulatory networks inference. IntOMICS is designed to be easily extended by another modality. The current implementation is tuned for gene expression, copy number variation, and DNA methylation data. However, the user can infer regulatory network, even if copy number variation or DNA methylation data (or both) are not available. IntOMICS is a powerful resource for exploratory systems biology and can provide valuable insights into biological processes' complex mechanisms that have a vital role in personalized medicine.

## AVAILABILITY

IntOMICS is open source software written in R, under GPL-2, and available at https://bioconductor.org/packages/IntOMICS. The implementation relies on existing R packages.

## References

[B1] Chen, Y., Li, Y., Xue, J., et al. 2016. Wnt-induced deubiquitination FoxM1 ensures nucleus b-catenin transactivation. EMBO J. 35, 668–684. 26912724 10.15252/embj.201592810 PMC4801947

[B2] Cooper, G.F. 1989. Current research directions in the development of expert systems based on belief networks. Appl. Stochast. Models Data Analysis. 5, 39–52.

[B3] Dai, W., Teodoridis, J.M., Zeller, C., et al. 2011. Systematic CpG Islands Methylation Profiling of Genes in the Wnt Pathway in Epithelial Ovarian Cancer Identifies Biomarkers of Progression-Free Survival. Clin. Cancer Res. 17, 4052–4062. 21459799 10.1158/1078-0432.CCR-10-3021 PMC3431504

[B4] Geiger, D., and Heckerman, D. 1994. Learning gaussian networks, 235–243. Proceedings of the 10th Conference on Uncertainty in Artificial Intelligence.

[B5] Hasin, Y., Seldin, M., and Lusis, A. 2017. Multi-omics approaches to disease. Genome Biol. 18, 83. 28476144 10.1186/s13059-017-1215-1 PMC5418815

[B6] Kang, M., Ko, E., and Mersha, T.B. 2022. A roadmap for multi-omics data integration using deep learning. *Brief*. Bioinform. 23, bbab454. 10.1093/bib/bbab454 PMC8769688 34791014

[B7] Lucas, P.J., van der Gaag, L.C., and Abu-Hanna, A. 2004. Bayesian networks in biomedicine and health-care. Artif. Intell. Med. 30, 201–214. 15081072 10.1016/j.artmed.2003.11.001

[B8] Madigan, D., York, J., and Allard, D. 1995. Bayesian graphical models for discrete data. Int. Stat. Rev. Revue Int. De Stat. 63, 215–232.

[B9] Neapolitan, R.E. 1990. Probabilistic Reasoning in Expert Systems: Theory and Algorithms. John Wiley & Sons, Inc., New York, NY, USA.

[B10] Ogata, H., Goto, S., Sato, K., et al. 1999. KEGG: Kyoto Encyclopedia of Genes and Genomes. Nucleic Acids Res. 27, 29–34. 9847135 10.1093/nar/27.1.29 PMC148090

[B11] Pačínková, A., and Popovici, V. 2022. Using empirical biological knowledge to infer regulatory networks from multi-omics data. BMC Bioinformatics. 23, 351. 35996085 10.1186/s12859-022-04891-9 PMC9396869

[B12] Pearl, J. 1988. Probabilistic Reasoning in Intelligent Systems: Networks of Plausible Inference. Morgan Kaufmann Publishers Inc., San Francisco, CA, USA.

[B13] Subramanian, I., Verma, S., Kumar, S., et al. 2020. Multi-omics data integration, interpretation, and its application. Bioinform. Biol. Insights. 14, 1–24. 10.1177/1177932219899051 PMC7003173 32076369

[B14] The Cancer Genome Atlas Research Network. 2011. Integrated genomic analyses of ovarian carcinoma. Nature. 474, 609–615. 21720365 10.1038/nature10166 PMC3163504

[B15] Zhang, W., Klinkebiel, D., Barger, C.J., et al. 2020. Global DNA hypomethylation in epithelial ovarian cancer: Passive demethylation and association with genomic instability. Cancers. 12, 764. 32213861 10.3390/cancers12030764 PMC7140107

